# Improved clinical outcomes after arthroscopic partial rotator cuff repair with rotator interval shift for the treatment of massive irreparable anterior L-shaped rotator cuff tears

**DOI:** 10.1186/s13018-025-05975-x

**Published:** 2025-06-03

**Authors:** Yu-Sheng Chen, Fa-Chuan Kuan, Wei-Ren Su, Chih-Kai Hong, Yueh Chen, Kai-Lan Hsu

**Affiliations:** 1 Department of Orthopedics, Taipei Municipal Hospital YangMing Branch, Taipei, Taiwan; 2 Department of Orthopaedic Surgery, National Cheng Kung University Hospital, College of Medicine, National Cheng Kung University, Tainan, Taiwan; 3 Skeleton Materials and Bio-Compatibility Core Lab, Research Center of Clinical Medicine, National Cheng Kung University Hospital, College of Medicine, National Cheng Kung University, Tainan, Taiwan; 4 Department of Orthopedics, Kaohsiung Veterans General Hospital Tainan Branch, Tainan, Taiwan

**Keywords:** Rotator cuff tear, Massive, Irreparable, L-shaped, Rotator cuff repair, Rotator interval, Acromiohumeral interval

## Abstract

**Purpose:**

The treatment of massive anterior L-shaped rotator cuff tears (RCTs) remains challenging. This study focused on restoration of humeral head coverage by superiorly repositioning the rotator interval tissue. The study aimed (1) to present the short-term clinical and radiological outcomes of arthroscopic partial repair combined with rotator interval shift and (2) to identify risk factors associated with failure in restoring superior humeral head coverage.

**Method:**

A retrospective review was conducted on arthroscopic rotator cuff repairs performed by a single surgeon between January 2018 and December 2022. Patients with irreparable anterior L-shaped tears who underwent partial repair and rotator interval shift and had a follow-up of 2 years were included. The measured outcomes included range of motion, pain, and functional scores. Humeral head coverage was evaluated using magnetic resonance imaging (MRI) at 6 months postoperation, and patients were classified as either healed or having a retear. Logistic regression analysis was conducted to identify factors associated with retears.

**Results:**

The study included 45 patients (19 men and 26 women) with an average age of 66.53 ± 6.70 years. After 2 years, significant improvements were observed in forward elevation (from 77.73 ± 40.96 to 156.11 ± 25.89), abduction (from 82.89 ± 43.78 to 161.33 ± 22.90), American Shoulder and Elbow Surgeons scores (from 43.24 ± 15.77 to 83.16 ± 10.27), and Constant–Murley scores (from 44.98 ± 15.76 to 86.38 ± 10.02; all *p* < 0.001). Visual analog scale pain scores also decreased (from 7.09 ± 2.35 to 1.29 ± 1.70). MRI results at 6 months showed that 30 of 45 patients (66.7%) had healed, while the retear rate was 33.3%. The acromiohumeral interval (AHI) was identified as the only factor significantly associated with retear. The odds ratio for predicting retear in patients with a preoperative AHI of < 5.0 mm was 5.50. (95% CI: 1.43–21.10, *p* = 0.013).

**Conclusion:**

Arthroscopic partial repair combined with rotator interval shift is an effective treatment option for irreparable anterior L-shaped RCTs, demonstrating favorable short-term clinical and radiological outcomes. However, patients with a preoperative AHI of less than 5 mm are at greater risk of retear, potentially leading to reduced postoperative range of motion.

**Level of evidence:**

Level IV, case series.

## Introduction

Rotator cuff tears (RCTs) are the most common upper extremity condition seen by primary care and orthopaedic surgeons [1]. Arthroscopic rotator cuff repair has emerged as the primary treatment for RCTs, demonstrating low retear rates and favorable clinical outcomes [2, 3]. However, the management of massive, irreparable RCTs remains challenging, as severe tendon retraction, fatty degeneration, and involvement of multiple tendons are often indicators of irreparability [4, 5]. Tendon retraction often limits the possibility of successful repair to the bony footprint, and repairs performed under high lateralizing tension are associated with a high failure rate [6]. In addition, the anterior L-shaped RCTs, distinguished by a longitudinal split through the rotator interval and retraction at the anterolateral corner of the supraspinatus (SSP) tendon [7], often progresses rapidly. This accelerated progression is attributed to high stress concentration at the base of the split [8], which exacerbates tear enlargement and increases tension during repair. As a result, these tears are associated with a higher likelihood of repair failure.

To address these challenges, various treatment strategies have been proposed, including partial repair, patch augmentation [9, 10], superior capsular reconstruction [11, 12], tendon transfer [13, 14] and ballon spacer [15, 16]. However, the latter three approaches often require the use of allografts or synthetic grafts, which may increase surgical complexity and overall healthcare costs. Burkhart et al. introduced partial repair as a treatment option for irreparable RCTs in 1993 by considering a “suspension bridge” analogy [17, 18]. Subsequently, numerous techniques for partial repair of the SSP, including margin convergence [19] and interval slides [20–23], have been developed, offering alternatives that can be employed before resorting to salvage procedures such as reverse total shoulder arthroplasty or tendon transfers.

Modern theories regarding shoulder biomechanics have indicated that the SSP muscle acts as a stabilizing “roof” over the humeral head, preventing upward displacement and maintaining a stable center of rotation for the glenohumeral joint [24]. The rotator interval, as described by Neer, is located between the SSP and subscapularis (SSC) muscles [25]. This region includes the coracohumeral ligament (CHL), the superior glenohumeral ligament (SGHL), the glenohumeral capsule, and the biceps tendon [26]. The primary role of the rotator interval is to stabilize the glenohumeral joint by restricting excessive external rotation and limiting posterior and inferior movement [27].

This study focused on restoration of humeral head coverage in cases of massive anterior L-shaped RCTs by superiorly repositioning the rotator interval tissue. The aim of this study was (1) to present the short-term clinical and radiological outcomes following arthroscopic partial repair combined with rotator interval shift and (2) to identify risk factors associated with retear. The hypothesis of this study was that arthroscopic partial repair combined with rotator interval shift would effectively restore superior anatomy and lead to improved clinical outcomes.

## Method

### Study population

This retrospective case series was approved by our institution’s institutional review board. Patients who had undergone arthroscopic rotator cuff repair performed by a single surgeon (W.-R.S.) were retrospectively recruited from a medical center in southern Taiwan, with the procedures conducted between January 2018 and December 2020. Patients with irreparable anterior L-shaped RCT who underwent arthroscopic partial repair combined with rotator interval shift and had detailed surgical records available for the first two postoperative years were included. An irreparable anterior L-shaped RCT is defined by (1) a massive tear greater than 5 cm in the anteroposterior dimension [28], involving two or more tendons with anterior extension into the rotator interval; and (2) the inability to achieve full tendon coverage of the footprint, even after sufficient release intraoperatively. The exclusion criteria were as follows: (1) a history of previous shoulder surgery; (2) preoperative signs of shoulder joint osteoarthritis, such as glenohumeral joint narrowing; and (3) a follow-up period of < 2 years.

### Surgical method

The surgery was typically performed under general anesthesia with the patient positioned in the lateral decubitus posture. A 3-point traction system (Arthrex, Naples, FL, USA) was employed to maintain arm traction throughout the procedure. Aseptic preparation and draping were performed, and a standard posterior portal was established. Subsequently, a standard anterior portal was created using spinal needle localization, placed just lateral to the tip of the coracoid process. The glenohumeral joint space was then examined and debrided as necessary. In cases where a superior labral anterior–posterior lesion or partial tear of bicep long head were present, a biceps tenotomy was typically performed without any additional procedures. For Lafosse grade II or higher SSC tears, repair was performed using a 70° arthroscope and posterior retraction of the humeral head for better visualization. After releasing surrounding tissue, a double-loaded anchor was placed at the footprint. A polydioxanone suture was used to shuttle braided sutures through the tendon, creating two mattress sutures from distal to proximal [29]. 

**Fig. 1:**
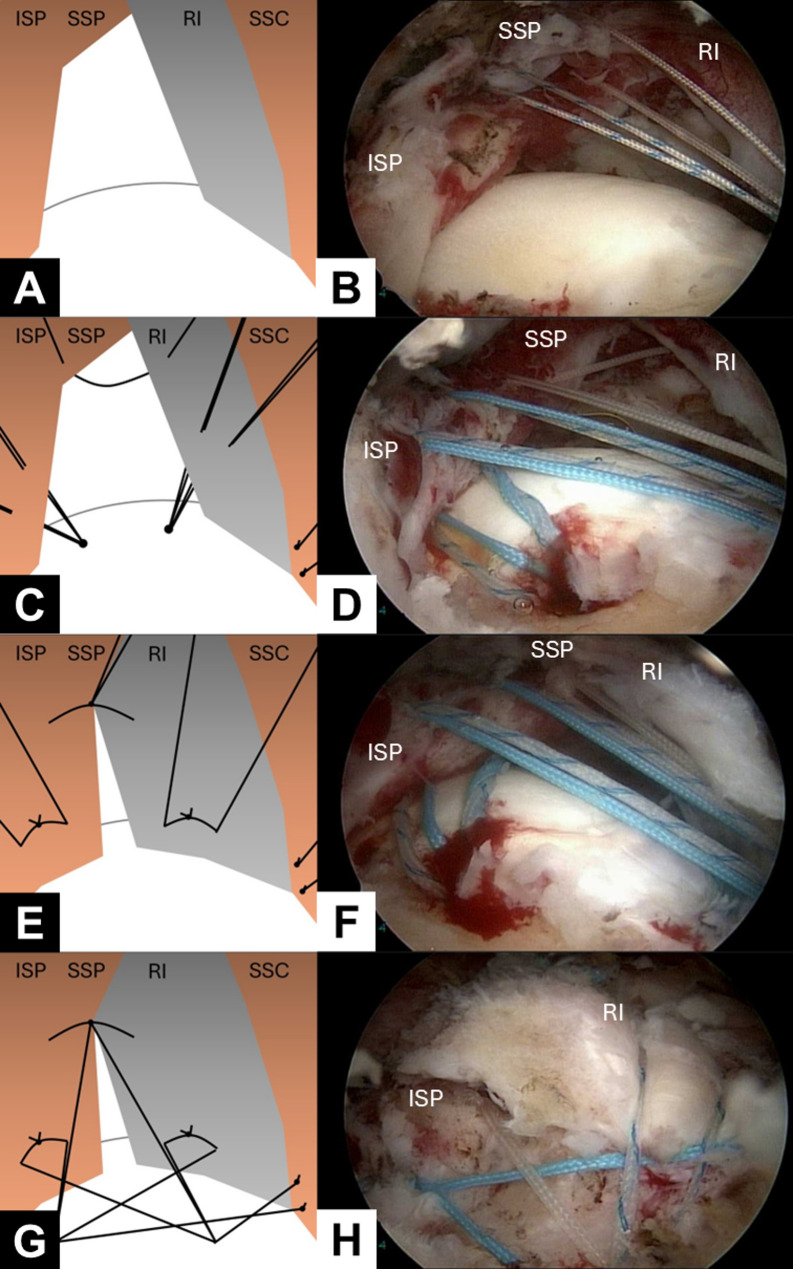
Illustrative and arthroscopic images depicting the procedure of arthroscopic partial repair with rotator interval shift. The patient was positioned in the lateral decubitus position, with traction applied to the right arm. The arthroscope was introduced through the posterolateral viewing portal. (1) Check reparability: Traction sutures were placed on the supraspinatus (SSP); however, reduction to the footprint was unsuccessful (**A, B**). (2) Anchor placement: Two suture anchors were inserted into the footprint, and the sutures from both anchors were passed through the rotator interval (RI) and infraspinatus (ISP) (**C, D**). (3) Suture management: Additional side-to-side repairs were performed between the RI and ISP (**E, F**). (4) Lateral row placement: Following suture management, the suture limbs from the subscapularis (SSC), RI, ISP, and side-to-side repairs were pulled laterally over the footprint to form a suture bridge by using two knotless anchors (**G, H**). (ISP, infraspinatus; SSC, subscapularis; SSP, supraspinatus; RI, rotator interval)

The arthroscope was then repositioned to visualize the subacromial space, and additional anterolateral and lateral portals were established using a spinal needle. Subsequently, bursectomy and limited acromioplasty were performed. Traction sutures were passed through the SSP and infraspinatus (ISP), and attempts were made to achieve primary repair to the footprint. If the SSP tendon could not be adequately repaired to the footprint, a rotator interval shift technique was employed. Under arthroscopic view, the rotator interval appears as a sling-like band extending from the coracoid process to the greater tuberosity, situated anterior to the SSP footprint (Fig. 1A and B). Unlike the rotator interval slide technique described by Lo et al. [23], which involves releasing the origin of the interval, our technique mobilizes the rotator interval posteriorly to cover the residual footprint that the retracted SSP is unable to reach. To perform rotator interval shift technique, the footprint of the rotator cuff was prepared, and two double-loaded suture anchors were inserted at the articular margin of the greater tuberosity. The first anchor was placed in the anteromedial aspect of the footprint, adjacent to the biceps groove. The second anchor was positioned 1.5–2 cm posterior to the first, in the posterior aspect of the footprint. The sutures from these anchors were passed through the rotator interval and ISP by using an antegrade suture passer (Arthrex; Fig. 1C and D). Additional side-to-side repairs were performed between the rotator interval and ISP tendons. Depending on the size of the gap, one or two side-to-side sutures were placed (Fig. 1E and F). Subsequently, the rotator interval and ISP tendon were secured to the footprint with mattress sutures. After suture management, the suture limbs from the knots on the SSC, rotator interval, ISP, and side-to-side repair were pulled laterally down to the lateral aspect of the footprint. This formed a suture bridge, which was secured using two knotless anchors (Fig. 1G and H).

### Clinical and radiographic evaluation

For clinical evaluation, both preoperative and postoperative assessments were conducted, with data collected from routine clinical visits prior to surgery and at 2 years postoperatively. The range of motion (ROM) was measured on the basis of the degree of forward elevation and abduction. Clinical outcomes were evaluated using the American Shoulder and Elbow Surgeons (ASES) score and the Constant–Murley scoring system. Additionally, shoulder pain was assessed using the visual analog scale (VAS).

For radiographic evaluation, standardized MRI examinations were conducted both preoperatively and at 6 months postoperation. The MRI scans were independently reviewed by two orthopedic shoulder specialist surgeons who were not involved in the study. SSP was assessed using T2-weighted oblique coronal scans by applying the Patte classification [30]. In this system, stage 1 indicates that the retracted stump is located near the bone, stage 2 denotes that it is at the level of the humeral head, and stage 3 signifies that it is at the level of the glenoid or higher.

To assess SSC tears, we employed the Lafosse classification [31], based on both preoperative images and intraoperative findings. In this classification, a Lafosse grade I tear is considered stable. Lafosse grades II and III indicate tears affecting more than one-third and two-third of the upper part of the tendon, respectively. Lafosse grades IV and V represent complete tears.

The degree of fat infiltration in the SSP and ISP muscles was assessed using the most lateral MRI image where the spine of the scapula intersects with the coracoid process (Y-view image). The Goutallier classification system was applied [32], with classifications ranging from grade 0 to 4. Grade 0 indicates no fat infiltration, grade 1 indicates some fatty streaks, grade 2 indicates noticeable fat but less fat than muscle, grade 3 indicates equal amounts of fat and muscle, and grade 4 indicates more fat than muscle.

The degree of atrophy in the SSP was evaluated using Y-view images [33]. In this assessment, muscle atrophy is classified into three grades: grade 1 indicates that the muscle is positioned above the tangent line, grade 2 indicates that the muscle just touches the tangent line, and grade 3 indicates that the muscle is clearly below the tangent line.

This study exclusively focused on massive RCTs, and therefore, it employed the acromiohumeral interval (AHI) and Hamada classification as key evaluation tools. The AHI was measured on plain radiographs. The Hamada classification [34], which is based on AHI measurements, categorizes the severity of cuff tear arthropathy. In this system, grade 1 indicates an AHI > 6 mm, grade 2 indicates an AHI = 5 mm, grade 3 indicates a reduced AHI and acromial acetabularization, grade 4 involves additional narrowing of the glenohumeral joint, and grade 5 indicates humeral head collapse.

**Fig. 2:**
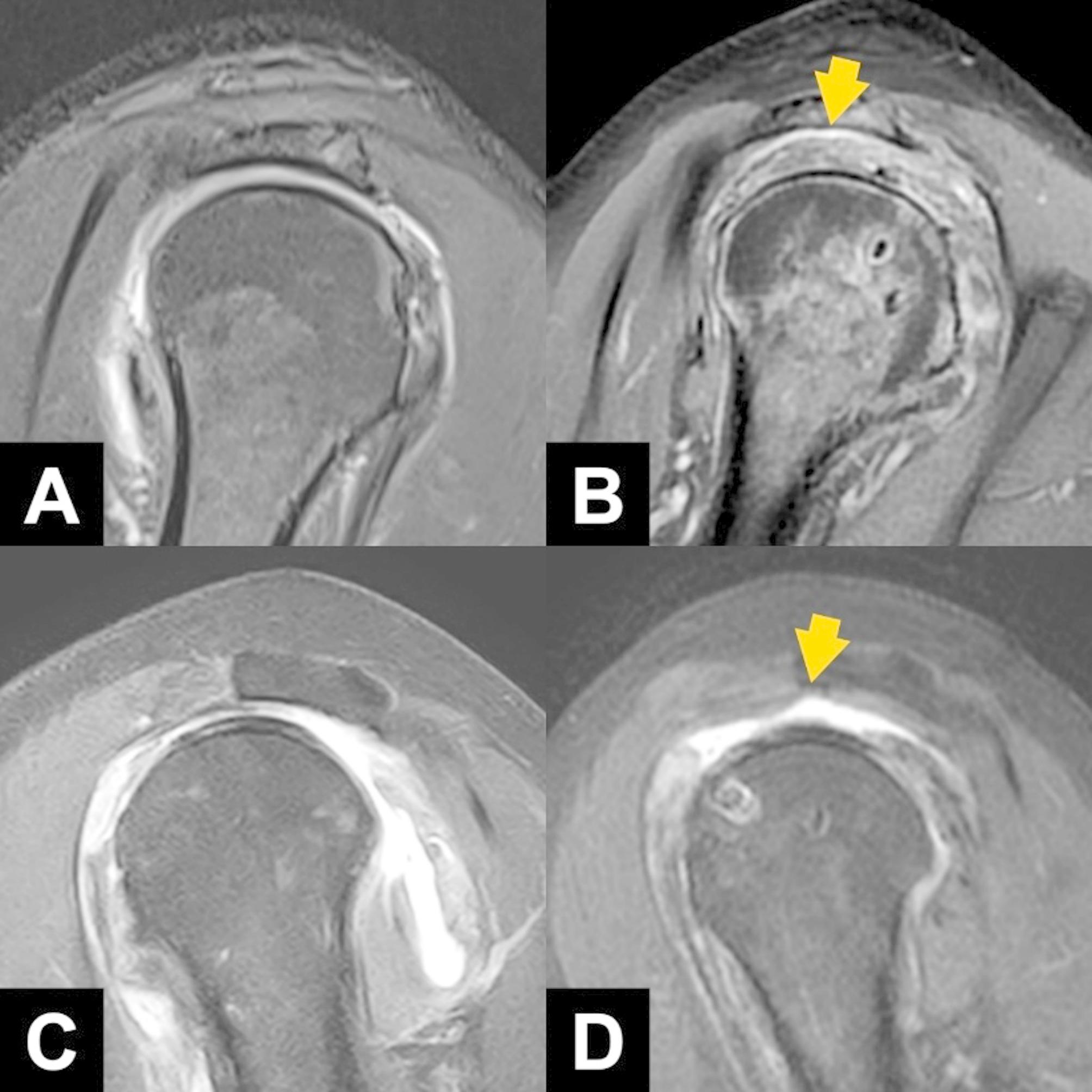
Assessment of soft tissue coverage over the humeral head by using sagittal views from magnetic resonance imaging. In the “headed” group, the preoperative (**A**) and postoperative (**B**) images revealed complete soft tissue coverage over the highest point of the humeral head (arrow). Conversely, in the “retear” group, the preoperative (**C**) and postoperative (**D**) images revealed a lack of coverage (arrow)

In follow-up MRI evaluations, retears of the repaired RCTs were classified according to the Sugaya classification system, which involves five types: type 1 involves sufficient tendon thickness with uniformly low signal intensity, type 2 involves sufficient thickness with areas of partial high intensity, type 3 involves insufficient tendon thickness without discontinuity, type 4 involves the presence of a minor discontinuity, and type 5 involves a major discontinuity in the tendon. Furthermore, to determine whether the rotator interval shift effectively restored humeral head coverage, sagittal MR images were used to evaluate soft tissue coverage. The “healed” group included cases in which the soft tissue fully covered the highest point of the humeral head, indicating Sugaya types 1 to 3 (Fig. 2A and B). Conversely, the “retear” group included cases in which coverage was lacking, indicating Sugaya types 4 and 5, that is, retraction of the mobilized rotator interval and failure of the interval shift procedure (Fig. 2C and D).

### Postoperative rehabilitation

After surgery, the patient was instructed to wear an abduction brace for 4–6 weeks, during which time no ROM exercises were permitted. Subsequently, gentle passive ROM exercises were gradually introduced, with the exercises typically involving supine forward elevation or internal rotation by using a towel. Strengthening exercises were initiated approximately 3 months postoperation. Patients were advised to refrain from engaging in sports and heavy labor activities until 6 months after the operation. Thereafter, the patients were gradually permitted to resume their regular activities and manual work. To ensure consistency and minimize any potential biases in the rehabilitation process, a standard postoperative rehabilitation protocol was adhered to.

### Statistical analysis

The study employed the paired *t* test and chi-square test to analyze preoperative and postoperative clinical and radiographic data, namely the Constant score, ASES score, and ROM. The paired *t* test was used for assessing continuous data, whereas the chi-square test was employed for assessing categorical data. Additionally, patients were categorized into two groups: those with intact sagittal coverage (healed) and those with a lack of sagittal coverage (retear). A comparative analysis of demographic, clinical, and radiographic data was conducted between these groups. Although multiple outcome measures were analyzed, no formal adjustment for multiple comparisons was made, as this was an exploratory analysis. To determine the minimal clinically important difference (MCID) using the distribution-based approach, we applied half (0.5) of the standard deviation of the data. A logistic regression analysis was conducted to identify independent variables influencing healing. Subsequently, the Youden index was applied to determine the optimal cut-off point for the covariates. All statistical analyses were performed using IBM SPSS, version 23.0 (IBM, Armonk, NY, USA), with two-tailed *p* values of < 0.05 considered significant.

## Results

**Fig. 3:**
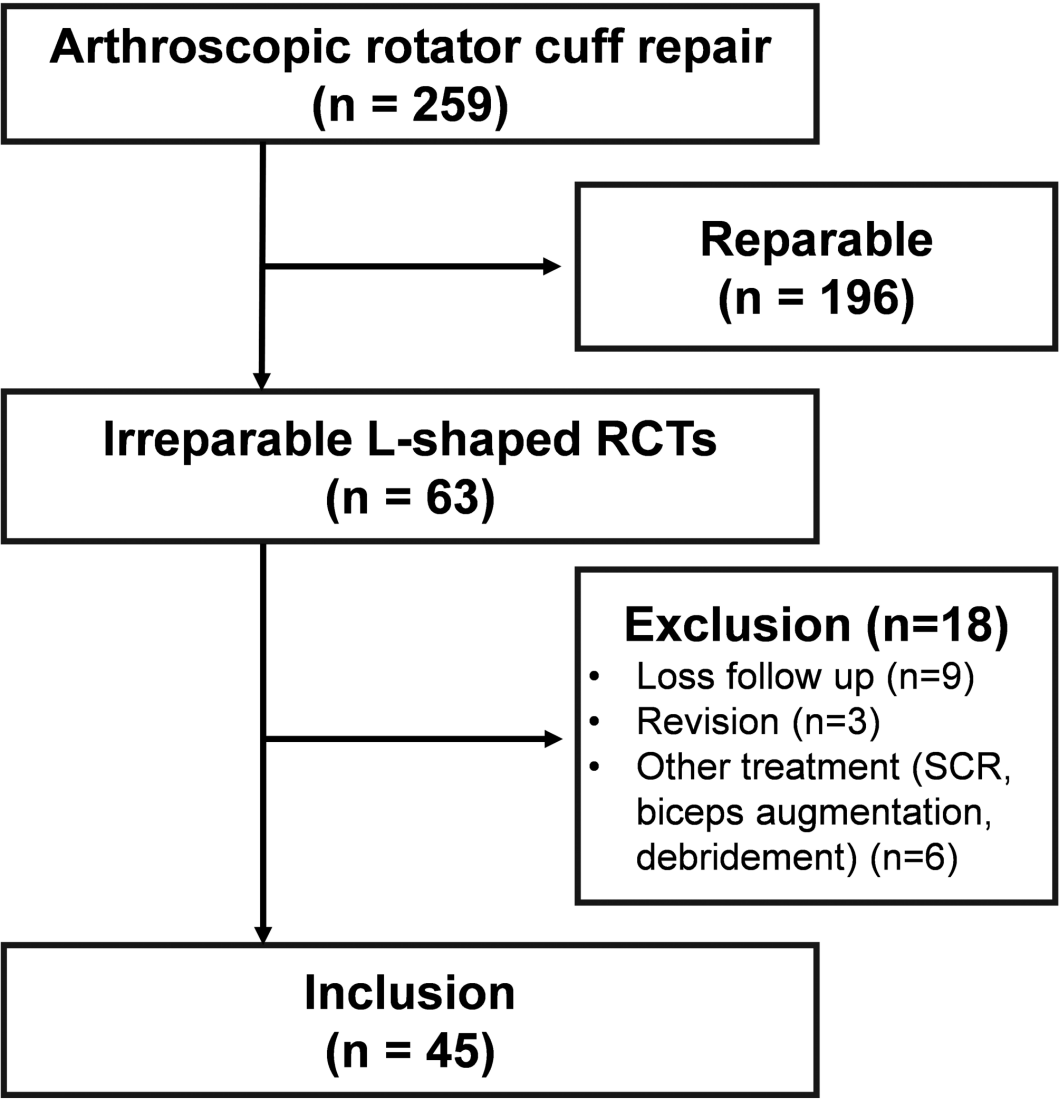
Flowchart illustrating the enrollment and inclusion of patients in the study. (RCTs, rotator cuff tears; SCR: superior capsule reconstruction)

**Fig. 4:**
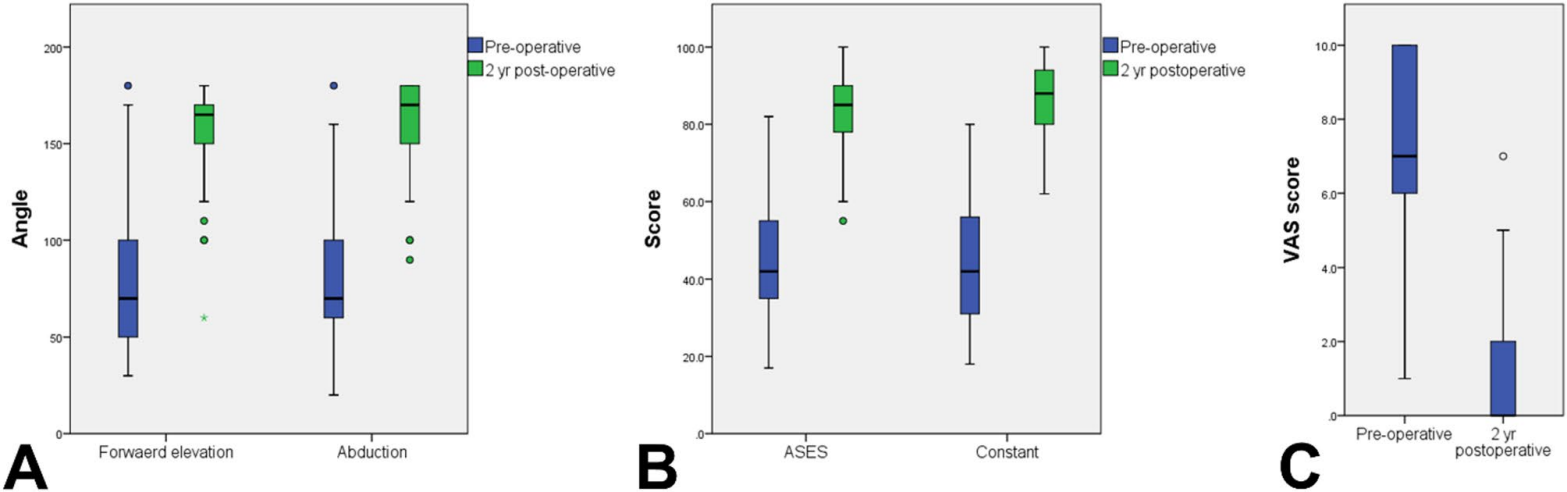
Comparison of preoperative and 2-year postoperative outcomes: (**A**) angles of forward elevation and abduction; (**B**) ASES score and Constant–Murley score; (**C**) VAS score. (ASES, American Shoulder and Elbow Surgeons; VAS, visual analog scale)

Of the 63 patients with irreparable L-shaped RCTs who underwent arthroscopic treatment, 45 were enrolled in the study, as shown in the flowchart in Fig. 3. The cohort comprised 19 men (42.2%) and 26 women, with a mean age of 66.53 ± 6.70 years (range, 52–70 years). The mean AHI was 5.56 ± 1.84 mm (range, 2.77–9.47 mm). Among the participants, 25 (55.69%) were classified as having a Patte III SSP tear. The demographic data and preoperative MRI findings are summarized in Table 1. After a follow-up period of 2 years, significant improvements were noted in various clinical parameters: forward elevation increased from 77.73 ± 40.96 to 156.11 ± 25.89 (*p* < 0.001), abduction improved from 82.89 ± 43.78 to 161.33 ± 22.90 (*p* < 0.001), the ASES score increased from 43.24 ± 15.77 to 83.16 ± 10.27 (*p* < 0.001), and the Constant–Murley score increased from 44.98 ± 15.76 to 86.38 ± 10.02 (*p* < 0.001). Additionally, a significant reduction was noted in the VAS pain score, from 7.09 ± 2.35 to 1.29 ± 1.70 (*p* < 0.001; Fig. 4A and B, and 4C). No complications, such as revision surgery, wound infection, nerve injury, or anchor dislodgement, were observed during follow-up.

**Table 1:** Demographic data and preoperative MRI findings for the included cases

Case number	45
Age (y/o)	66.53 ± 6.69
Gender (Men/Women)	19/26
Side (Right/Left)	33/12
AHI (mm)	5.56 ± 1.84
Hamada grade (1/2/3/4/5)	20/18/6/1/0
Patte classification of SSP tear (1/2/3)	0/20/25
Lafosse classification of SSC tear (1/2/3/4/5)	3/10/25/7/0
Goutallier classification of SSC (1/2/3/4)	11/32/1/1
Goutallier classification of SSP (1/2/3/4)	0/29/13/3
Goutallier classification of ISP (1/2/3/4)	1/28/16/0
Atrophy of SSP (1/2/3)	7/26/12

AHI, acromiohumeral interval; ISP, infraspinatus; MRI, magnetic resonance imaging; SSC, subscapularis; SSP, supraspinatus

After 6 months of follow-up during which MRI scans were conducted, 30 of the 45 patients (66.7%) demonstrated soft tissue coverage of the humeral head and were classified as “healed.” The remaining 15 patients (33.3%) were classified as “retear.” Preoperative demographic and MRI data revealed a significantly lower AHI in the retear group than in the healed group (4.69 ± 1.51 mm vs. 5.99 ± 1.86 mm, *p* = 0.031; Table 2**)**. Postoperation, patients in the healed group exhibited significantly greater improvements in both forward elevation and abduction compared with those in the retear group (forward elevation: 88.67° ± 8.30° vs. 57.80° ± 36.37°, *p* = 0.015; abduction: 91.83° ± 45.42° vs. 51.67° ± 40.16°, *p* = 0.006). However, the improvements and achievements of MCID in ASES, Constant–Murley, VAS scores were comparable between the two groups **(**Tables 3 and 4**)**.

**Table 2:** Demographic data and preoperative imaging findings for comparison of the healed and retear groups

	Healed (*N* = 30)	Retear (*N* = 15)	*p*-value
**Demographic**			
Age	66.93 ± 6.64	65.73 ± 6.97	0.547
Gender (M: F)	11:19	8:7	0.286
Side (R: L)	22:8	11:4	1.000
Follow up (m)	25.1 ± 4.1	27.1 ± 6.2	0.211
**Preoperative image**			
AHI	5.99 ± 1.86	4.69 ± 1.51	**0.031**
Hamada grade (1:2:3:4)	15:12:3:0	5:6:3:1	0.337
Lafosse classification (1:2:3:4:5)	3:8:16:3:0	0:2:9:4:0	0.244
Patte grade of SSP (1:2:3)	0:16:14	0:4:11	0.090
Goutallier classification of SSC (1:2:3:4)	9:20:1:0	2:12:0:1	0.274
Goutallier classification of SSP (1:2:3:4)	0:19:10:1	0:10:3:2	0.344
Goutallier classification of ISP	1:21:8:0	0:7:8:0	0.185
Atrophy of SSP (1:2:3)	6:16:8	1:10:4	0.484
**Intraoperative procedure**			
Treatment of biceps long head (In situ: tenotomy or tear)	8:22	5:10	0.642

AHI, acromiohumeral interval; ISP, infraspinatus; SSC, subscapularis; SSP, supraspinatus

**Table 3:** Postoperative radiographic and clinical outcomes for comparison of the headed and retear groups

	Healed (*N* = 30)	Retear (*N* = 15)	*p*-value
**Sugaya classification (1:2:3:4:5)**	**0:11:19:0:0**	**0:0:0:3:12**	**< 0.001**
**Forward elevation**			
Pre-operative	75.00 ± 39.81	83.20 ± 44.07	0.568
2 years follow-up	163.67 ± 18.43	141.00 ± 32.20	**0.005**
*p*-value	**< 0.001**	**0.001**	
Improved	88.67 ± 8.30	57.80 ± 36.37	**0.015**
**Abduction**			
Pre-operative	75.67 ± 41.91	97.33 ± 45.27	0.127
2 years follow-up	167.50 ± 18.89	149.00 ± 25.79	**0.002**
*p*-value	**< 0.001**	**0.001**	
Improved	91.83 ± 45.42	51.67 ± 40.16	**0.006**
**VAS for pain**			
Pre-operative	7.03 ± 2.51	7.20 ± 2.08	0.835
2 years follow-up	1.17 ± 1.29	1.53 ± 2.10	0.674
*p*-value	**< 0.001**	**0.001**	
Improved	5.87 ± 3.15	5.67 ± 2.58	0.611
**ASES Score**			
Pre-operative	42.50 ± 14.94	44.73 ± 17.78	0.800
2 years follow-up	84.23 ± 8.77	81.00 ± 12.83	0.477
*p*-value	**< 0.001**	**0.001**	
Improved	41.73 ± 17.11	36.27 ± 19.25	0.335
**Constant-Murley score**			
Pre-operative	42.10 ± 14.27	50.73 ± 17.47	0.145
2 years follow-up	86.87 ± 8.30	85.40 ± 13.08	0.942
*p*-value	**< 0.001**	**0.001**	
Improved	44.77 ± 16.76	34.67 ± 22.28	0.145

ASES: American Shoulder and Elbow Surgeons; VAS: Visual analog scale

**Table 4:** Rates of achievement of minimal clinically important difference (MCID) between the headed and retear groups

	Healed (*N* = 30)	Retear (*N* = 15)	*p*-value
**Achieved MCID**			
ASES	93.3 (28/30)	86.7 (13/15)	0.459
VAS for pain	80.0 (24/30)	93.3 (14/15)	0.245
Constant-Murley score	93.3 (28/30)	73.3 (11/15)	0.063

ASES: American Shoulder and Elbow Surgeons; MCID: Minimal clinically important difference; VAS: Visual analog scale

**Fig. 5:**
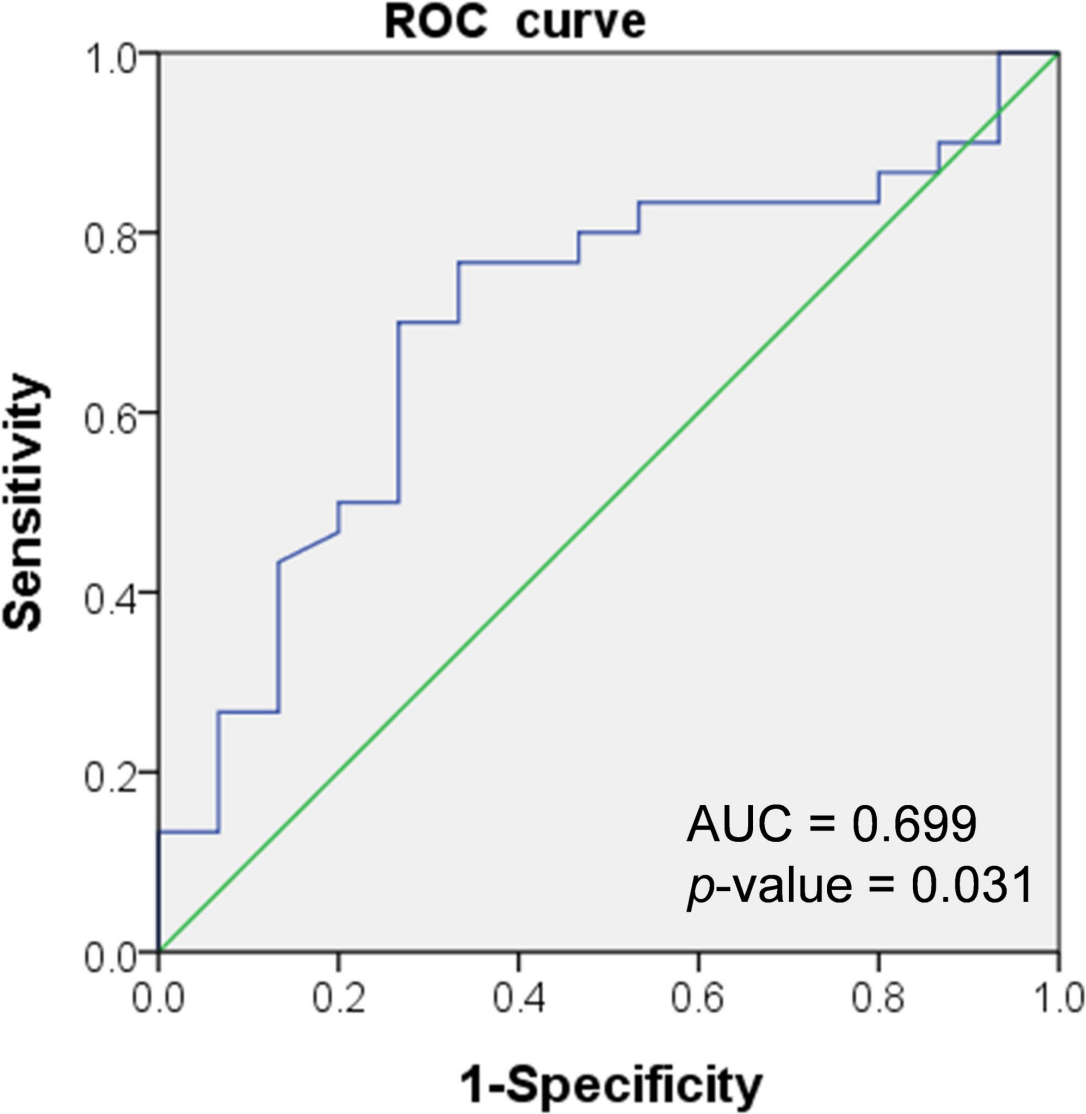
Receiver operating characteristic (ROC) curve demonstrating that an AHI of < 5 mm can be used to predict retear following arthroscopic partial repair and rotator internal shift. (AHI, acromiohumeral interval, AUC, Area under the curve)

Comparative analysis indicated that the AHI was the only factor associated with the retear. A cutoff value of 5.0 mm was determined using the Youden index. The ROC curve used to predict healing yielded an area under the curve (AUC) of 0.699. (Fig. 5). With a cutoff point of 5.0 mm for AHI, the sensitivity and specificity were determined to be 0.733 and 0.667, respectively. The odds ratio for predicting retear in patients with an AHI of < 5.0 mm was 5.50 (95% CI: 1.43–21.10, *p* = 0.013).

## Discussion

The main finding of this study is that arthroscopic partial repair combined with rotator interval shift effectively improves functional scores and range of motion in irreparable anterior L-shaped RCTs over two years. However, a preoperative AHI of less than 5 mm is associated with a higher risk of retear, potentially limiting postoperative range of motion.

Massive RCTs pose a considerable challenge for shoulder surgeons. Although advancements in arthroscopic techniques have considerably enhanced the ability to repair most RCTs, the management of massive tears remains difficult because they often involve retracted tendons and compromised quality in surrounding tissue. In certain cases, extensive release of soft tissue and the capsule may allow mobilization of severely retracted rotator cuff tears. However, for anterior L-shaped tears, it can be difficult to achieve adequate approximation of the lateral tendon edge to the medial rotator cuff footprint. Loss of the anterior margin of the rotator cable in L-shaped tears can compromise its stress-shielding function, leading to increased strain and potentially further tear propagation [35].

In cases of irreparable anterior L-shaped RCTs where complete repair is unattainable, arthroscopic partial repair has emerged as a viable option. Various studies have demonstrated that this approach can lead to significant improvements in postoperative shoulder function and ROM. Berth et al. [36] reported that arthroscopic partial rotator cuff repair yields more favorable functional outcomes than debridement alone. Additionally, research by Godeneche et al. [37] and Iagulli et al. [38] have revealed that both partial and complete repairs of massive RCTs result in comparable functional improvements during short- to mid-term follow-up periods. However, over the long term, the functional outcomes associated with partial repairs tend to be inferior to those associated with complete repairs [39], though both approaches yield significant improvements relative to baseline conditions [40]. Notably, the long-term efficacy of partial rotator cuff repairs may be compromised by problems related to repair integrity. Studies have reported retear rates following partial repairs ranging from 48–53% [36, 37, 41]. This high incidence of retears can adversely affect the durability and effectiveness of the repair over time, leading to suboptimal long-term results.

Structured augmentation of partial repairs is critical to ensuring effective management of irreparable RCTs, especially in anterior L-shaped tear. The long head of the biceps (LHB) tendon has been identified as a viable option for enhancing partial repairs. Savarese et al. [42], Llinas et al. [43], Chiang et al. [44], and Laprus et al. [45] have demonstrated that arthroscopic partial repair with LHB tendon augmentation leads to more favorable healing and functional outcomes than does partial repair alone [46]. However, Jeon et al. [47] have found a similar clinical result between biceps augmentation and partial tendon repair in treating massive anterior L-shaped RCTs. This finding suggests that biceps augmentation may not serve effectively as a functional tendon or significantly impact the restoration of stable fulcrum kinematics. Furthermore, this technique is not suitable for cases where the LHB tendon is ruptured or significantly affected by tendinopathy.

Alternative autografts, such as fascia lata [48] and the semimembranosus tendon [30], have also been used for augmentation. However, these procedures are technically challenging, and their use has been reported in only a few cases. Additionally, various allografts and synthetic materials have been employed for augmentation, including dermal patches [49, 50], biological collagen [51], and scaffolds [52]. However, the use of graft augmentation increases operative time and costs [53]. Furthermore, the introduction of foreign materials involves inherent risks, including risks of infection, inflammation, and graft degradation [54].

The rotator interval, a triangular structure located in the anterosuperior aspect of the glenohumeral joint, plays a crucial role in shoulder stability [55]. This interval comprises the CHL, SGHL, glenohumeral capsule, biceps tendon, and fibers of both the SSC and SSP muscles [26]. Collectively, these dense fibrous structures provide resistance to inferior and posterior translation of the humeral head, maintain negative intraarticular pressure, and stabilize the LHB[13, 36]. The rotator interval is also referred to as the “comma sign” or “comma tissue” [56].

The rotator interval has been utilized to assist in RCT repairs. Lo and Burkhart [23] introduced the “interval sliding” technique, which involves releasing the rotator interval’s origin from the coracoid process to enhance rotator cuff mobility. Corpus at al. [20] applied this technique in subscapularis repairs and reported excellent clinical outcomes. Dilisio and Neyton proposed that incorporating the comma tissue into the repair process helps achieve stable fixation of both the upper border of the SSC and the anterior rotator cable of the SSP [57]. Furthermore, Hackl et al. [58] revealed that stabilizing the comma sign leads to superior biomechanical properties compared with single-row repair in cadaveric studies. These findings underscore the efficacy of using the rotator interval for augmentation in RCT repairs [59], with this benefiting not only the SSC but also the SSP.

In this study, the rotator interval was shifted posteriorly to cover the footprint inadequately addressed by the SSP, without releasing its proximal attachment. This approach preserves tissue continuity and stability, differing from the interval slide technique [20–23]. Despite its thin appearance, the rotator interval demonstrated sufficient integrity to reinforce the anterior margin not reached by the SSP, offering both mechanical and potential biological benefits. Given that Jeong et al. [22] reported similar retear rates even after aggressive release, we chose to shift the tissue rather than release it to maintain structural integrity. The short-term follow-up results indicated promising functional improvements. However, approximately one-third of the patients exhibited a lack of soft tissue coverage over the tip of the humeral head in the sagittal plane, indicating a nonhealed cuff or superior capsule. Notably, the retear rate was lower than that previously reported for partial repairs alone (48–53%) [36, 37, 41]. However, the incidence of retear was significantly higher in patients with a reduced AHI. Goutallier et al. established that a narrow AHI is associated with full-thickness ISP tears [60], whereas Furuhata et al. demonstrated a strong correlation between ISP integrity and humeral head migration [61]. These findings indicate that the healing status of the ISP plays a critical role in the success of the rotator interval shift procedure. A well-healed ISP reduces footprint exposure, acting not only as a mechanical buffer between the acromion and humeral head [47] but also providing the biological foundation needed for rotator interval healing. Notably, the severity of an ISP tear can be predicted using the AHI. Therefore, a lower AHI may serve as a predictor of poor healing following the rotator interval shift procedure.

Although a retear was observed in approximately one-third of the patients, improvements in shoulder function were still noted. This finding aligns with previous literature indicating that functional outcomes do not always correlate with structural integrity [62–64]. However, Jeong et al. [65] reported that retears are significantly associated with the progression of osteoarthritis and may lead to a decline in functional outcomes at least five years postoperatively. In the present study, patients with retears continued to demonstrate improved shoulder function; however, a decline in range of motion, particularly in abduction and forward elevation, was observed. A longer follow-up period may provide further insight into the long-term effects of retear on shoulder function.

### Limitations

This study has several limitations that should be acknowledged. This was a single-center, retrospective study with a limited sample size, and the follow-up period was relatively short, which prevented us from conducting a comprehensive assessment of the potential for retears. Future studies should include extended follow-up periods and additional imaging evaluations. Additionally, the use of the rotator interval to cover the humeral head complicates accurate assessments of healing. Although the presence of sagittal coverage may serve as an indication of healing, the quality and biological characteristics of the tissue remain unclear.

## Conclusion

Arthroscopic partial repair combined with rotator interval shift appears to be effective in the short term for treating irreparable anterior L-shaped RCTs; it is associated with promising short-term clinical and radiological outcomes. However, patients with a preoperative AHI of less than 5 mm are at greater risk of incomplete sagittal plane coverage, potentially leading to reduced postoperative range of motion. These results are limited to the short term, and further research is needed to assess long-term outcomes.

## Data Availability

No datasets were generated or analysed during the current study.

## Abbreviations

AHI: Acromiohumeral interval
ASES: American Shoulder and Elbow Surgeons
AUC: Area under the curve
CHL: Coracohumeral ligament
ISP: Infraspinatus
LHB: Long head of the biceps
MCID: Minimal clinically important difference
MRI: Magnetic resonance imaging
RCTs: Rotator cuff tears
RI: Rotator interval
ROC: Receiver operating characteristic
ROM: Range of motion
SGHL: Superior glenohumeral ligament
SSC: Subscapularis
SSP: Supraspinatus
VAS: Visual analog scale
